# 伴BCR::ABL1 e6a2融合基因慢性髓性白血病1例报告并文献复习

**DOI:** 10.3760/cma.j.cn121090-20230930-00156

**Published:** 2024-03

**Authors:** 静静 李, 玲玲 岳, 鹏云 曾, 重阳 吴, 慧玲 陈

**Affiliations:** 兰州大学第二医院血液科，兰州 730030 Department of Hematology, the Second Hospital of Lanzhou University, Lanzhou 730030, China

## Abstract

e6a2转录本类型的慢性髓性白血病（CML）临床上非常罕见，通常与疾病的侵袭有关，其临床特点及与酪氨酸激酶抑制剂疗效间的关系尚不明确。本文通过回顾性分析1例以全身多发溶骨性骨质破坏并胃肠道、淋巴结等多器官嗜酸性粒细胞浸润为特征的e6a2融合基因阳性CML患者的临床特点及相关实验室检查，并进行相关文献复习。该患者Ph染色体阳性，伴+8染色体，CML常见BCR::ABL1转录本均阴性，但通过RT-PCR检测出e6a2转录本阳性表达，予以达沙替尼100 mg/d治疗，3个月后复查达到了完全血液学反应、完全细胞遗传学反应及分子学反应4.0。e6a2转录本在临床上非常罕见，需研究更多e6a2转录本的病例以明确其临床特点，并改善这些罕见病例的治疗效果。

## Abstract (en)

Chronic myeloid leukemia（CML）with e6a2 transcript type is very rare in clinic,which is usually related to disease aggressiveness. Its clinical characteristics and relationship with tyrosine kinase inhibitor efficacy are still unclear. In this paper, the clinical characteristics and related laboratory tests of a patient with e6a2 fusion gene positive CML characterized by multiple osteolytic bone destruction throughout the body and eosinophil infiltration in gastrointestinal tract, lymph nodes and other organs were retrospectively analyzed, and the relevant literature was reviewed. The patient was Ph chromosome positive with chromosome +8, and the common BCR::ABL1 transcript of CML was negative, but e6a2 transcript was positive detected by RT-PCR. The patient was treated with dasatinib 100 mg/d. Three months later, the patients achieved CHR, CCyR and MR4.0. However, the e6a2 transcript is very rare in clinical practice, and more cases of e6a2 transcript need to be studied to clarify its clinical characteristics and improve the treatment effect of these rare cases.

慢性髓性白血病（CML）是一种来源于造血干细胞的骨髓增殖性肿瘤，其中90％的患者以t（9;22）（q34;q11）导致BCR和ABL基因融合为特征[1]–[3]。多数患者为Ph染色体阳性CML，表达e13a2（b2a2）或e14a2（b3a2）BCR::ABL1 mRNA。其他位点的表达非常罕见，BCR与ABL外显子a3（e13a3、e14a3、e1a3）融合的CML偶见报道，其他相对罕见的BCR与ABL转录本还有e2a2、e6a2。不同转录本类型的CML，对伊马替尼的治疗反应存在差异，这些转录本对预后和治疗的影响尚有争议[4]–[5]。我们回顾性分析了1例以全身多发溶骨性骨质破坏并胃肠道、淋巴结等多器官嗜酸性粒细胞浸润为特征的CML患者，该患者Ph染色体阳性，伴+8染色体，CML常见BCR::ABL1转录本均阴性，但检出罕见e6a2转录本，现报道如下。

## 病例资料

患者，男，34岁，主因“胸背部疼痛20 d，加重4 d”于2023年8月22日就诊于我院骨科。既往无哮喘、过敏等病史。否认家族遗传病病史。查体：轻度贫血貌。颈部及双侧腋窝多发淋巴结肿大，最大约鹌鹑蛋大小，质韧，活动度好，无压痛。心肺未见异常。肝、脾肋缘下未触及。四肢肌力及肌张力均正常。实验室检查：血常规：WBC 21.28×10^9^/L，中性粒细胞占62％，嗜碱性粒细胞占17％，中性粒细胞绝对计数13.11×10^9^/L，嗜碱性粒细胞绝对计数3.57×10^9^/L，RBC 4.29×10^12^/L，HGB 118 g/L，PLT 320×10^9^/L。全身骨显像（SPECT）示:颅骨多处、多个椎体、骶骨、左侧髂骨多发溶骨性骨质破坏，代谢异常增高，考虑多发性骨髓瘤可能。胸椎及腹部CT示：左侧多发肋骨、胸腰骶椎及左侧髂骨多发骨质破坏。胃镜检查示：胃黏膜病变，胃隆起型病变；慢性萎缩性胃炎（C2型）并糜烂十二指肠球炎。病理活检示：（胃窦、胃体大弯）黏膜慢性炎症，急性炎症活动期（中性粒细胞：+++），间质内较多嗜酸性粒细胞浸润，>25个/HPF，不排除嗜酸性胃炎。肠镜检查示：回盲瓣溃疡、直肠炎。病理活检示：（回盲瓣）黏膜慢性炎症，急性炎症活动期，间质水肿，可见嗜酸性粒细胞浸润，不排除嗜酸性胃肠炎的可能。经血液科会诊后完善骨髓穿刺、骨髓活检、基因及染色体等检查。骨髓细胞形态学检查示：增生极度活跃，粒系比例偏高，粒细胞颗粒增多增粗。嗜酸性粒细胞比例偏高，占30％。红系、巨核细胞系等未见明显异常。外周血细胞形态学分析示：WBC升高，粒细胞比例偏高，嗜碱性粒细胞比例偏高。意见：①多考虑CML；②髓系肿瘤伴嗜酸性粒细胞增多?考虑血液系统疾病，转入我科。完善腹部彩超示：脾指数：29.6 cm，脾肋缘下约2.8 cm，脾大。骨髓免疫分型示：在CD45/SSC点图上设门分析，淋巴细胞约占0.5％，比例明显降低。原始区域细胞约占0.5％，分布散在。单核细胞约占0.5％，比例明显降低。嗜酸性粒细胞约占16.0％，比例增高。中性粒细胞约占78.5％，比例增高，部分细胞考虑存在发育异常。提示：嗜酸性粒细胞比例增高。骨髓活检+网银染色示：MF-1级，造血组织增生较活跃，嗜酸性粒细胞比例增高，考虑骨髓增殖性肿瘤（MPN）。骨髓FISH检测示：FGFR1异常（3F82％）。PDGFR、BCR::ABL1（P190、P210、P230）及MPN等基因均阴性。骨科、放射科、风湿免疫科、呼吸科及病理科等多学科讨论意见：①为明确诊断需完善淋巴结及骨组织病理活检，以排除肿瘤骨转移或淋巴结转移；②需排除不典型或其他特殊类型的CML。腋窝淋巴结活检示：（腋窝）皮肤组织、皮下汗腺周围大量嗜酸性粒细胞浸润，考虑嗜酸性粒细胞增多症，不排除慢性嗜酸性粒细胞白血病。免疫组化染色示：CKp（上皮+），S-100（−），cD1a（−），Langerin（−），LCA（淋巴细胞+），Ki-67阳性细胞数30％。骨组织活检示：（T4椎体病变）送检组织为松质骨，骨髓腔正常结构破坏，为增生的纤维和大量不同发育的嗜酸性粒细胞及残余的骨髓细胞，诊断为骨内嗜酸性粒细胞增多症，又称金氏病（Kimura病）。免疫组化染色示：嗜酸性粒细胞示S100（−），langerin（−），CD21（−），CD68（−），cDla（−），Ki-67阳性细胞数<1％，支持上述诊断。患者的染色体核型分析示：46，XY，t（9;22）（q34:q11.2）[15]/47，idem，+8[14]/46，XY[5]（图1）。因Ph染色体阳性，但CML常见融合基因均阴性，故采用RT-PCR的方法检测罕见型BCR::ABL1转录本，结果示BCR::ABL1罕见型e6a2阳性（图2），明确诊断为CML（慢性期，BCR::ABL1罕见型：e6a2），于2023年9月14日予以达沙替尼100 mg/d，口服。患者住院期间胸背部疼痛难忍，予以盐酸曲马多及酮铬酸镇痛效果不佳，故予以泼尼松50 mg（1 mg·kg^−1^·d^−1^）治疗后，患者疼痛缓解。现达沙替尼治疗近3个月，患者胸背部疼痛等症状完全缓解，查体脾脏未触及。血常规提示：WBC 8.8×10^9^/L，中性粒细胞占31％，嗜碱性粒细胞占1％，HGB 131 g/L，PLT 237×10^9^/L。骨髓细胞形态学检查提示：骨髓增生活跃，粒系比例正常，骨髓中原始细胞占3％，CML治疗后骨髓象。考虑达到完全血液学反应（CHR）。染色体核型分析：46，XY[7]，Ph^+^细胞为0，达到完全细胞遗传学反应（CCyR）。e6a2融合基因转为阴性，定量检测：BCR::ABL1 e6a2/ABL1 为0.00％（以<0.01％作为标准），达到分子学反应4.0（MR4.0）。

**图1:**
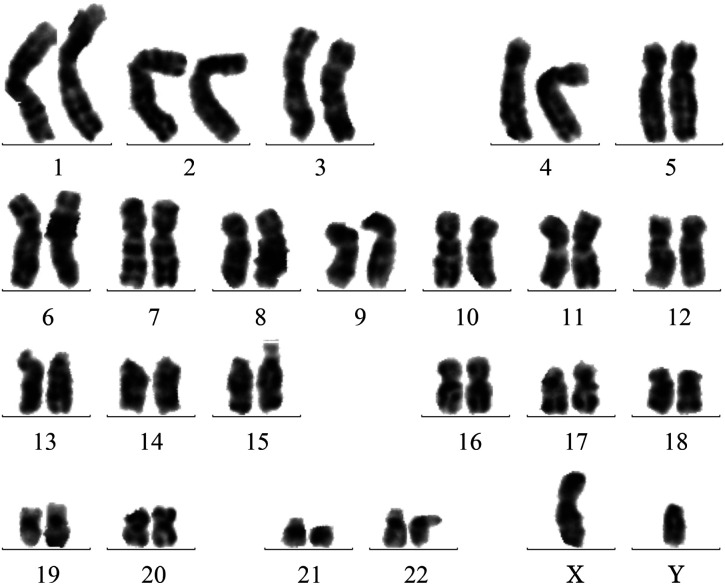
本例伴BCR::ABL1 e6a2融合基因慢性髓性白血病患者染色体核型分析

**图2:**
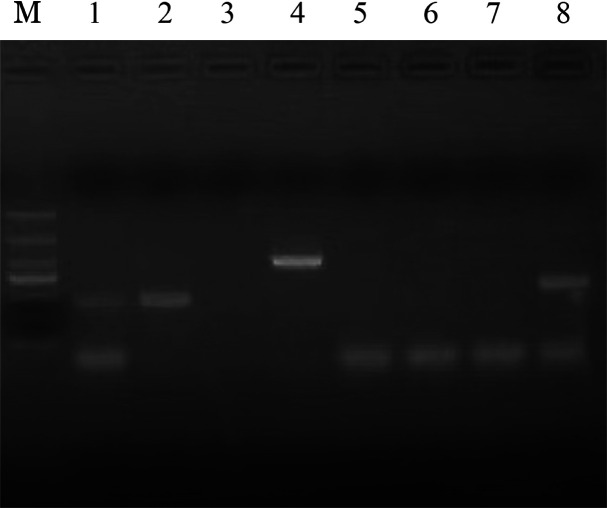
RT-PCR检测本例慢性髓性白血病患者e6a2转录本表达 M：1 000 bp Marker；1、3～7：e6a2阴性患者；2：本例e6a2阳性患者；8：阳性对照（P210型阳性）

## 讨论及文献复习

e6a2转录本非常罕见，其编码的蛋白质尽管结构域接近P190蛋白激酶，主要与CML的侵袭性过程相关，易误诊为急性白血病。迄今为止，关于伴e6a2转录本的CML患者结局的资料有限。有文献报道，其致癌能力可被伊马替尼抑制。一些病例报告提示侵袭性病程，对伊马替尼[6]–[7]和达沙替尼[7]治疗耐药。但目前关于第二代酪氨酸激酶抑制剂（TKI）一线治疗这一基因型的疗效报道较少[8]。2005年，Roti等[9]首次报道e6a2和e1a2 BCR::ABL1阳性CML，该病例主要的临床特点为持续发热、视野盲点和胸骨痛。其5′ABL1和3′BCR在单独克隆中存在隐性缺失，且缺失的der（9）的基因组区域不同。这两种缺失均在整个监测过程中被检测到。使用伊马替尼400 mg/d治疗，在治疗后的21个月随访中，该患者的疾病稳定。截至2011年已报道的12例e6a2 BCR::ABL1 CML患者中，男性11例。其中7例死亡，5例病情缓解。其中3例仅接受伊马替尼治疗；其余2例在allo-HSCT后缓解[7]。Zagaria等[10]报道的1例e6a2转录本表达的CML病例，其以具有许多异常颗粒的早期嗜酸性粒细胞为特征，对达沙替尼的治疗反应较好。Manzella等[8]报道的1例BCR::ABL e6a2融合转录本的慢性期CML患者接受常规剂量尼洛替尼一线治疗（300 mg，2次/d），随访结果未知。Ramdohr等[11]报道2例e6a2融合转录本的慢性期CML患者，认为第二代TKI尼洛替尼对表达不典型e6a2 BCR::ABL1融合转录本的患者有效。

我们中心报道的这例伴e6a2融合基因CML，是1例34岁年轻男性患者，以胃、肠、淋巴结及骨骼等多器官嗜酸性粒细胞浸润为特征，脾脏触诊未见肿大，结合患者血常规、骨髓细胞形态及初次有关CML及嗜酸性细胞增多症等相关基因筛查，考虑嗜酸性细胞增多症可能性较大。但患者染色体核型分析回报提示Ph染色体阳性，伴染色体数目异常（即出现了+8染色体）。+8是最常见的染色体数目异常，它既可作为一个单独的染色体畸变出现，也可伴随其他染色体异常同时出现，并与白血病的临床表现、分型、演进和预后密切相关，常预示着病情的演进和预后不佳[12]–[13]。此外，该患者FISH结果提示FGFR1异常（3F82％），重排阴性（重排的信号为1R1G1F，而该患者的FGFR1信号表现为1R1G3F，是由于+8造成的）。鉴于以上结果，我们再次与伴嗜酸性粒细胞增多和FGFR1重排髓系肿瘤[14]、8p11骨髓增生异常综合征等进行鉴别。8p11骨髓增生异常综合征是一种罕见的同时具有外周血白细胞计数明显增高、骨髓中髓系细胞增生、嗜酸性粒细胞增多、BCR::ABL1（−）、淋巴瘤、白血病转化特征的临床综合征，类似CML或慢性粒-单核细胞白血病骨髓象，极易误诊。髓系肿瘤伴FGFR1重排伴嗜酸细胞增多，是一种恶性侵袭性血液系统疾病，以髓系增生、嗜酸性细胞增多及FGFR1基因重排为特征，而本例患者缺乏FGFR1重排，与该类疾病亦不相符。因患者染色体核型分析提示Ph染色体阳性，我们高度怀疑罕见BCR::ABL1转录本可能，采用RT-PCR的方法对罕见BCR::ABL转录本进行定性检测，结果检测到e6a2转录本阳性表达，CML（慢性期，罕见型）诊断明确，予以达沙替尼 100 mg/d，治疗。根据以上复查结果，认为达沙替尼治疗疗效良好，继续口服达沙替尼 100 mg/d，定期随访。

其他罕见BCR::ABL1转录本类型CML病例也有相关报道，如McCarron等[15]报道1例使用Sanger测序法检测到e13a3转录本的CML患者，并认为使用伊马替尼可获得良好的细胞遗传学及分子生物学反应。表达e1a3转录本的患者，其临床病程发展缓慢，白细胞计数低，经伊马替尼治疗后可获得良好的细胞遗传学和分子生物学缓解。也有相关病例报道，e1a3 BCR::ABL1转录本类型的CML患者对伊马替尼反应良好，治疗后可迅速获得完全细胞遗传学缓解，但5个月后转为急性淋巴细胞白血病[16]。在TKI时代，罕见BCR::ABL1转录本的预后意义尚不确定。在一项回顾性研究中[17]，从由2 331例CML患者组成的队列中检出40例（1.7％）伴有罕见BCR::ABL1转录本。共检测出4种罕见转录本，分别为e1a2（0.9％）、e19a2（0.4％）、e13a3（0.1％）和e14a3（0.3％）。与具有典型转录本的患者相比，具有e1a2转录本的患者对TKI的反应较差，结局较差。e19a2转录本患者接受TKI治疗早期获得最佳反应率高，但多数患者因BCR::ABL1突变而失去CCyR，导致预后不良。携带e13a3/e14a3转录本的患者对TKI反应良好，预后良好。这些发现表明，TKI时代，在确定CML患者的治疗方案时，应考虑BCR::ABL1转录本的类型。

总之，由于CML罕见BCR::ABL1转录本类型的病例报道有限，缺乏诊疗共识，尚无法明确其临床特点，使得该类CML患者误诊率增加。在疑似CML患者中，需要考虑到罕见转录本的存在，对非典型BCR::ABL1融合转录本的CML患者进行必要的分子学检测至关重要[18]，包括FISH、二代测序（NGS）和Sanger测序法等，以防止误诊的可能性。此外，全面的测序还可能为非典型BCR::ABL1融合转录本及晚期CML患者的新型治疗确定靶点。第二代TKI，如达沙替尼，似乎对罕见转录本e6a2患者有效，要确定这些罕见转录本患者的最佳治疗选择，需要大宗病例的临床研究，尤其是多中心的临床试验。
